# Different types of oesophageal reconstructions in the contemporary era: a systematic review and network meta-analysis

**DOI:** 10.1007/s11845-025-04073-5

**Published:** 2025-08-29

**Authors:** Muireann Keating, Matthew G. Davey, William Murray, Eamon Franics, Noel E. Donlon

**Affiliations:** 1 Department of Plastics and Reconstructive Surgery, St. James’s Hospital, Dublin, Republic of Ireland; 2 Department of Surgery, Royal College of Surgeons in Ireland, Dublin 2, Dublin, Republic of Ireland; 3 Department of Surgery, Trinity St. James’s Cancer Institute, Trinity, St. James’s Hospital and Trinity College Dublin, Dublin, Republic of Ireland

**Keywords:** Anastomotic leak, Complications, Oesophageal cancer, Oesophageal reconstruction, Oesophagectomy

## Abstract

**Introduction:**

Oesophageal reconstruction is a complex operation that continues to present a surgical challenge associated with significant morbidity and its associated sequelae. The conventional gastric conduit remains the gold standard reconstructive technique when available. Alternative conduits for oesophageal replacement become necessary when the stomach is unavailable with common options for conduit creation being the jejunum and the colon.

The aim of this systematic review and network meta-analysis was to interrogate outcomes in oesophageal reconstruction with gastric pull-up, colonic interposition and jejunal flap.

**Methods:**

A systematic review of three electronic databases (PubMed, EMBASE and SCOPUS) was undertaken. An NMA as per the PRISMA-NMA guidelines. Statistical analysis was carried out using R and Shiny.

**Results:**

In a total of 19 studies, 3927 patients were included; 79.5% (3123/3927) of patients underwent gastric pull-up; 13.5% (531/3927) of patients underwent colonic interposition; 7% (273/3927) of patients underwent jejunal flap as their reconstructive method. At NMA, there was no significant difference in anastomotic leak rates, mortality rates, stricture formation, necrosis and length of stay between the three reconstructive techniques. Trend results showed jejunal flap performed better than colonic interposition in length of stay and mortality rates.

**Conclusion:**

At present, the gastric conduit is the conventional and first choice for oesophageal reconstruction ab initio after oesophagostomy. Colonic interposition and jejunal free flap represent viable options and are associated with non-inferior short-term surgical outcomes when gastric pull-up is not available or feasible.

## Introduction

Oesophageal cancers present significant challenges in management due to their complex anatomical location, organ involvement and functional demands [1]. The current overall 5-year survival for these malignancies is estimated to be between 5 and 20%, but this varies significantly according to site and stage [2, 3]. Traditional treatment methods include a multimodal approach with neoadjuvant chemo(radio)therapy and surgery which remains the mainstay of treatment. For the surgical management of these diseases, it is principally driven by Upper Gastrointestinal surgeons; however, increasingly multidisciplinary collaboration involving Otolaryngologists and Plastics and Reconstructive surgeons is paramount to successful outcomes for both oncological control and functional outcomes [1, 4]. The current mainstay treatment for early disease is surgical resection the extent of which is dependent on anatomical location of the primary tumour and may involve resection of the oesophagus as well as in some cases the pharynx and larynx, followed by reconstruction [4, 5].

Over the past 50 years, advances in microsurgical techniques have enabled the development of more complex and robust reconstructions [6]. Circumferential defects often require tubular reconstruction to restore gastrointestinal function and continuity, as well as in some cases pharyngeal and laryngeal reconstruction [1]. The spectrum of oesophageal reconstruction methods is broad, ranging from traditional options such as gastric pull-up to more contemporary approaches like jejunal interposition, colonic interposition, anterolateral thigh and radial forearm free flaps [7–10]. The gastric pull-up (GPU) operation is often the first-line reconstruction option for the oesophagus. The advantages of using the stomach as the conduit for oesophageal reconstruction include its robust vascular supply, ease of preparation and the need for only a single anastomosis for continuity [11]. However, there are cases where the GP reconstructive method may not be the most suitable option: cases of gastric tumour extension, corrosive gastric injury, previous gastric surgery or previously failed gastric pull-up procedures [12].

Colonic interposition (CI) and jejunal flap (JF) have gained traction as popular potential alternatives to the GP as an oesophageal substitute over recent years [8, 13]. Colonic interposition has been used for oesophageal reconstruction since the early 1990 s [14]. Either the right or left colon can be used as a conduit. The use of the jejunum as an oesophageal replacement was first described by Roux in 1907 [15]. Since then, there have been multiple described modifications using both pedicled and free-segmented techniques (REF). Each method carries its unique advantages and limitations, and the choice of reconstruction technique often depends on factors such as the extent of the resection, patient comorbidities, surgeon’s expertise and available subspecialties.

There is currently limited evidence and direct comparative studies on these various techniques. Given the critical nature of these procedures, a comprehensive understanding of the available reconstruction options is essential for optimizing patient outcomes. The aim of this systematic review and network meta-analysis was to interrogate early surgical outcomes in oesophageal reconstruction with gastric pull-up, colonic interposition and jejunal flap.

## Methods

A systematic review was performed in accordance with the Preferred Reporting Items for Systematic Reviews and Meta-analyses (PRISMA) extension statement for reporting of systematic reviews incorporating NMAs of healthcare interventions. This study was registered with the International Prospective Register of Systematic Reviews. Institutional ethical review board approval was not required for this review. No funding was obtained for this study. (PROSPERO ID: CRD42024577393).

### Search strategy

A systematic search was performed in January 2024 of three electronic databases (PubMed, EMBASE and Scopus). Each database was initially searched for relevant titles. This search included the following search terms: oesophagectomy, oesophageal cancer, gastric pull-up, jejunal flap and colonic interposition. Manual removal of duplicate studies was performed before all titles were screened. Covidence was used to facilitate tracking and management of screen abstracts. Studies considered to be appropriate had their abstracts and/or full text reviewed. Retrieved studies were reviewed to ensure inclusion criteria were met for a primary or secondary outcome at a minimum.

### Eligibility criteria

All published studies with full-text manuscripts comparing the outcomes of two or more, of gastric pull-up, jejunal flap and colonic interposition as a reconstruction method post oesophagectomy were included. The inclusion criteria for studies were as follows:

1. Studies of patients with malignant disease who underwent open or minimally invasive oesophagectomy, laryngopharyngooesophagectomy or pharyngooesophagectomy and immediate oesophageal reconstruction surgery
2. Studies that compared at least two of gastric pull-up, free jejunal flap and colon interposition reconstruction, with or without the supercharged procedure
3. Studies included only patients over 18 years old
4. Studies that included at least one of the following outcomes: anastomotic leak rate, length of stay, mortality or stricture formation
5. Studies that had full text articles
6. Studies written in the English language

Included studies were not restricted by year of publication.

The exclusion criteria were as follows:

1. Studies failing to fulfil the above inclusion criteria
2. Studies not published in the English language
3. Studies that included patients under the age of 18 years
4. Conference abstracts
5. Studies for which there was no full text available
6. Studies discussing outcomes in isolated pharyngeal reconstruction
7. Studies which discussed reconstruction outcomes in salvage procedures
8. Case reports and case series
9. Studies in which the primary indication for oesphagectomy and reconstruction was benign disease.

Search terms and search strategy can be found in the supplementary materials.

### Data extraction

Two authors (M. K and W. M) independently reviewed all studies collected through literature review. Covidence was used to maintain a collaborative database of each study. Both authors extracted the following data. Title and reference data including author, year of publication, digital object identifier (DOI), number of subjects, interventions under investigation and number treated by each intervention, gender and age of subjects and all outcome measures available in each study. After each stage of the reviewing process, the senior author (N. E. D) independently reviewed the selected literature and resolved any discrepancies in opinion.

### Statistical analysis

Descriptive statistics were used to outline the characteristics of the included studies. Data pertaining to anastomotic leak, stricture formation, mortality, graft necrosis and symptomatic reflux were expressed as dichotomous or binary outcomes, reported as odds rations (*OR*s) with 95% confidence intervals (*CI*s). *OR*s were calculated using crude event study data, to compare interventions using per-protocol data, where applicable. Continuous data were calculated using mean values, standard deviations (*SD*s) and pooled mean–variance with differences expressed as weighted mean differences (*WMD*s). Gastric pull-up (GPU) was the principal comparator for all analyses. Bayesian NMAs were conducted using meta meta [16]and Shiny packages [17] for R. Point estimates of effect sizes with a 95% *CI*. The results were considered statistically significant at the *p* < 0.05 if the 95% *CI*s did not include a value of 1. Estimates of mean and *SD*s were calculated from studies using standard statistical methods, where applicable [18, 19]. Rank probabilities were plotted against the possible ranks for all competing treatments. The confidence in estimates of the outcomes was assessed using the Confidence in Network Meta-Analysis (CINeMA) tool [20].

## Results

### Literature search

**Fig. 1:**
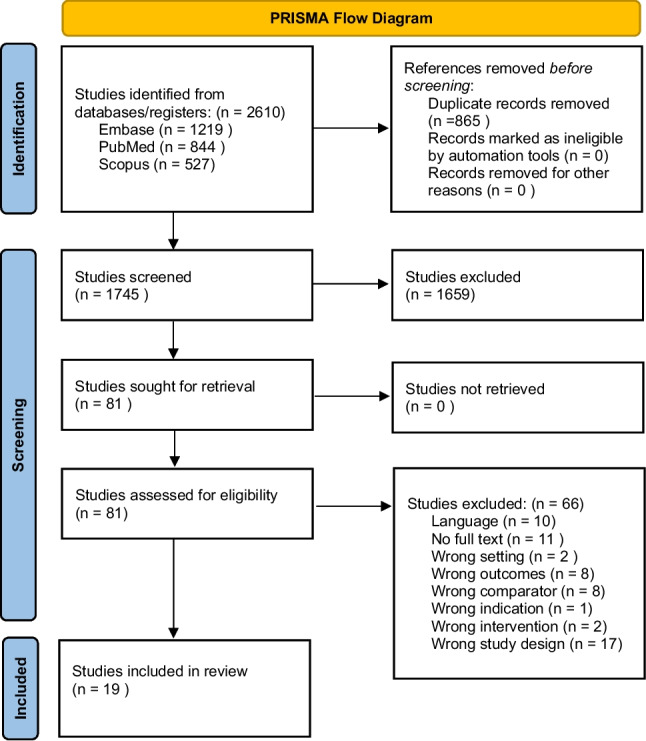
PRISMA flow diagram. Details of search results and screening process. Reasons for exclusion after full text screening are listed adjacent

In total, 2610 articles were identified, and 856 duplicate articles were excluded. After the removal of duplicates, study titles and abstracts were screened, resulting in 81 studies being eligible for full-text review. Of these, 19 studies met the eligibility criteria and were included. The PRISMA flow chart is illustrated in Fig. 1.

### Study characteristics

A total of 19 studies, including 3927 patients were included. The publication dates of the studies included were between 1991 and 2023. A total of 79.5% (3123/3927) of patients underwent gastric pull-up; 13.5% (531/3927) of patients underwent colonic interposition; 7% (273/3927) of patients underwent jejunal flap as their reconstructive method. The mean age at surgery was 61.5 years. Of the 2605 patients that the data was available for, 2154 (83%) were male, and 451 (17%) were female. Overall, 17/19 studies included gastric pull-up; 14/19 studies included colonic interposition; 10/19 included jejunal flap as a reconstruction method. There were 12/19 studies reported on isolated oesophageal reconstruction and 7/19 studies reported on pharyngeal +/− laryngeal +/− hypopharyngeal with oesophageal reconstruction. For all studies included, malignancy was the indication for resection and reconstruction. Table 1 illustrates a summary of all included studies.

**Table 1:** Summary of all patient data in included studies. *X* = not reported on in the study

Study, Year	Country	Study design	Study period	Multicentre	Recon method (*n*)	Supercharged	Anatomosis location	No of patients	Age (mean)	Male/female	Cancer stage	Pathology	Tumour location
Briel et al. [21]	USA	Retrospective	1996–2002	No	*GP* (230) *CI* (163)	X	Cervical	393	X	X	X	X	X
Carlson et al. [22]	USA	Retrospective	1970–1989	No	CI (19) *GP* (23) *JF* (26?	X	X	68	61	92/53	X	SCC (137) Salivary carcinomas (6) 2 papillary	Hypopharyngeal (100) Cervical oesophagus (45)
Chang et al. [23]	Taiwan	Retrospective	2012–2016	No	*GP* (15) *CI* (2) *GPJF* (6) *GPALT* (2)	X	Cervical	25	54	24/1	X	X	Hypopharyngeal Hypopharyngeal and oesophageal Hypopharyngeal and tongue Laryngeal and oesophageal
Daiko et al., 2007 [24]	Japan	Retrospective	1982–2002		*GP* (21) *JF* (50)	X	X	71	X	X	I: 6 II: 30 III: 38	SCC	Cervical
Davis et al. [25]	Hong Kong	Prospective	1982–2000	No	*GP* (959) *CI* (42)	No	Both	1001	62.7	840/161	0: 37 I: 48 II: 249 III: 553 IV: 113	Adeno: 107 SqCC: 873 Other: 21	Cervical: 52 Upper: 64 Middle: 503 Lower: 253 Cardia: 104 Double: 25
DeMeester et al. [26]	USA	Questionnaire	X	Yes	*GP* (116) *CI* (85)	X	Intra-thoracic	201	X	X	X	X	X
Doki et al. [27]	Japan	Retrospective	1998–2005	No	*CI* (28) *JF* (25)	Yes (Both)	Intra-thoracic	53	64.9	49/4	0: 4 I: 7 II: 17 III: 15 IV: 10	X	X
Elfeky et al. [28]	Eygpt	Retrospective	2007–2010	Yes	*GP* (33) PMMF (34) *JF* (37)	No	Cervical	104	59.5	65/39	T1: 6 T2: 11 T3: 7 N0: 17 N1: 7	SCC: All	Hypopharyngeal (80) Cervical oesophagus (24)
Ferahkose et al. [29]	Turkey	Retrospective	1996–2006	No	*GP* (38) *JF* (14)	No	Cervical	52	55.7	28/14	I: 8 II:9 III: 35	SCC: 47 Other: 5	Hypopharngeal: 19 Cervical: 33
Huttl et al. [30]	Germany	Questionnaire	1999	Yes	*GP* (653) *CI* (66)	No	Both	719	X	X	X	SqCC: 706 Barrets: 282	X
Iizuka et al. [31]	Japan	Retrospective	2000–2018	No	*GP* (57) *CI* (39)	X	X	96	63.6	84/12	X	X	X
Jiang et al. [32]	China	Retrospective	2009–2021	No	*CI* 11 *JF* 5	Yes (JF)	Both	16	64	14/2	I: 5 III: 9 IV: 2	Adenocarcinoma	Upper thoracic: 1 Mid: 2 Lower: 13
Kohl et al. [33]	Belgium	Retrospective	1990–1998	No	*GP* (92) *CI* (38)	No	Both	130	60 12	103/27	I: 21 II: 51 III: 52 IV: 6	Adeno: 62 SqSCC: 28 Cardia: 33	Upper: 14 Middle: 49 Lower: 33 Cardia: 34
Luan et al. [34]	USA	Retrospective	2004–2014	No	*GP* (85) *CI* (4) *JF* (15)	Yes (JF)	X	104	62.4	87/17	X	X	X
Stephens et al. [35]	USA	Questionnaire	2009–2013	No	*GP* (31) *JF* (14)	No	Both	45	60.6	31/14	X	Adeno: 26 Benign: 2 Other: 8	X
Sun et al. [36]	China	Retrospective	1988–2011	No	*GP* (48) *CI* (19)	No	Cervical	67	56.1	60/7	X	All SqSCC	Cervical: 56 Hypopharynx-oesophagus junction
Triboulet et al. [37]	France	Retrospective	1982–1999	No	*GP* (127) *CI* (5) *JF* (77)	X	Cervical	209	55	193/16		X	Hypopharyngeal: 131 Cervical: 78
Van Heijl [38]	Netherlands	Prospective	1996–2006	No	*GP* (593) *CI* (14)	No	Cervical	607	63	463/138	X	Adeno: 439 SCC: 439	X
Woods et al. [39]	Ireland	Retrospective	2008–2018	No	*GP* (12) *JF* (16)	X	Cervical	28	61.9	21/7	X	Adenocystic: 1 SCC: 29	Cervical oesophagus3 Other: 25

### Primary outcomes

#### Anastomotic leak

Overall, 15/19 studies (78.9%) reported on anastomotic leak. The overall anastomotic leak rate was 10.2% (315/3100). *JF* reconstruction showed the highest anastomotic leak rate at 18.1% (43/237), followed by *CI* at 12.4% (57/460) and *GP* at 8.9% (215/2403). Despite *JF* being associated with the highest anastomotic leak rate, there was no significant difference observed in anastomotic leak rates between the three reconstructive methods. League rank tables ranked *GP* best in terms of anastomotic leak.

### Secondary outcomes

#### Mortality

Eleven of the nineteen studies (58%) reported on in-hospital mortality. The overall in-hospital mortality rate was 7.7% (190/2473). *JF* had the lowest mortality rate at 1.8% (3/166), followed by *GP*% and *CI*%). At NMA, *JF* had a lower mortality rate when compared with *GP* (*OR* 0.15, 95% *CI* 0.17; 1.57). *CI* showed a higher mortality rate when compared with *GP* (*OR* 1.49, 95% *CI* 0.90; 2.47). However, these results were not statistically significant.

#### Stricture

Overall, 13/19 (68.4%) studies reported on stricture formation post oesophageal reconstruction. The overall stricture formation rate was 22% (619/2819). *CI* had the lowest stricture formation rate of 8.2% (31/380), followed by *JF* at 11.8% (19/161) and *GP* at 25% (569/2278). No significant difference between stricture formation rates and the reconstructive methods was identified. League rank tables ranked *CI* best in terms of stricture formation.

### Length of stay

In total, 10/19 studies (52.6%) reported on hospital length of stay. The mean length of stay was 25.3 days (*SD* 21.7). *CI* had the longest length of stay (34 *SD* 33.4), followed by *JF* (28 *SD* 21.7) and *GP* (24 *SD* 19.2). Using *GP* as a comparator, NMA failed to demonstrate a significant difference between the reconstructive options in the duration of hospitalisation. Compared to *GP*, *JF* had the largest mean difference − 1.28 (95% *CI* − 5.47;2.92), followed by *CI* (MD − 0.65 95% *CI* − 6.10; 4.81). League rank tables ranked *JF* best in terms of length of stay.

### Graft necrosis

**Fig. 2:**
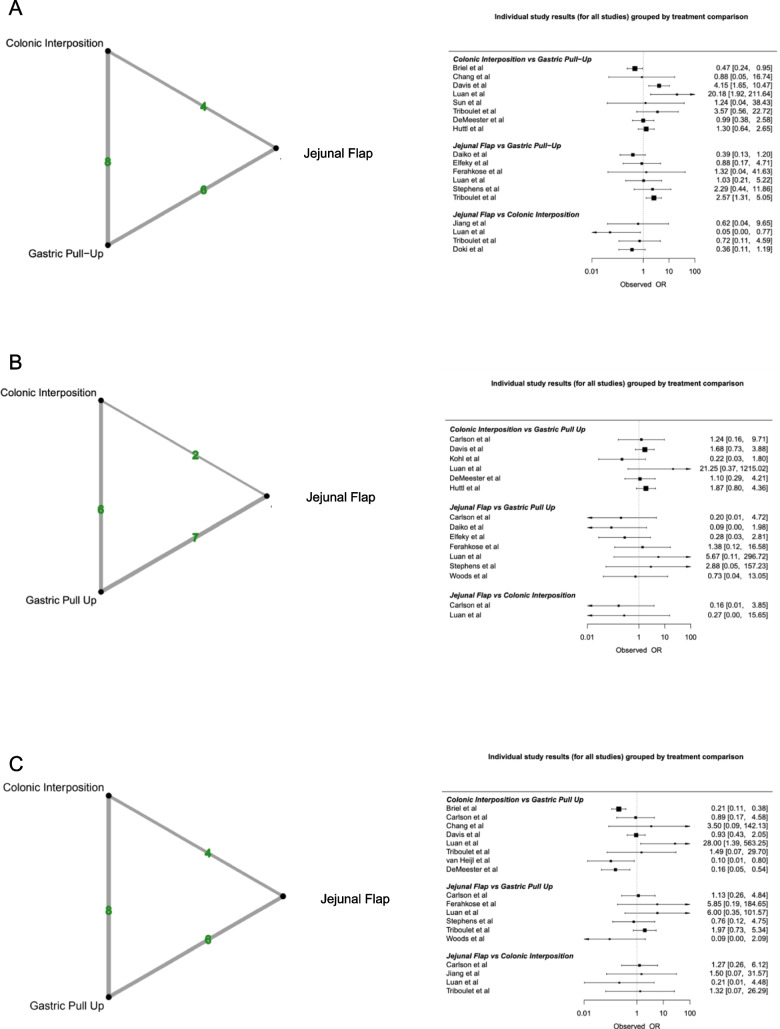

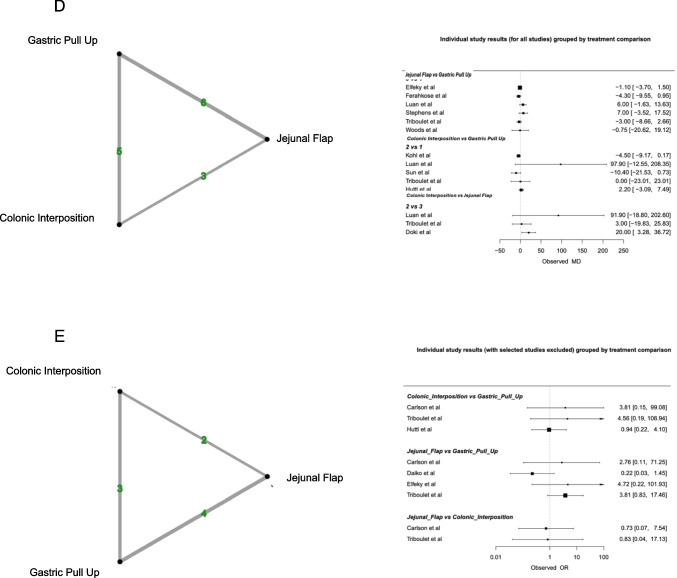
Network plots and data summary of all included individual studies assessing rates of **A** anastomotic leak, **B** mortality, **C** stricture formation, **D** length of stay and **E** graft necrosis

Five of the nineteen studies reported on graft necrosis (26.3%) (Fig. 2). The overall rate of necrosis was 3.4% (39/1135). *GP* had the lowest rate of graft necrosis at 3% (26/855), followed by *CI* at 3.3% (3/90) and *JF* at 5.3% (10/190). Compared to *GP*, both *CI* (*OR* 1.49, 95% *CI* 0.35; 6.38) and *JF* (*OR* 1.36, 95% *CI* 0.40; 4.63) showed a higher necrosis rate. However, at NMA, there was no statistically significant difference in graft necrosis between the 3 reconstructive options. League rank tables ranked *GP* best in terms of graft necrosis.

## Discussion

Our NMA included 19 retrospective studies, with a total of 3927 patients. NMA demonstrated that colonic interposition and jejunal free flap were associated with non-inferior surgical outcomes, where gastric pull-up was not available. This confirms our hypothesis that both colonic interposition and jejunal free flap are valid options in oesophageal reconstruction when the stomach is unavailable or in cases of recurrence. There were no statistically significant differences in the outcomes assessed, but several trends were identified. At NMA, *JF* had advantages over *CI* in terms of in-hospital mortality (*OR* 0.15), overall length of stay (28 *SD* 21.7) and necrosis (*OR* 1.36). *CI* did show an advantage compared to other reconstruction methods in terms of stricture formation (*OR*) but was inferior concerning other secondary outcomes. These trends are in keeping with the findings of our literature review. A notable finding from the review is the haphazard reporting of patient characteristics, complications and variation across the studies. Anastomotic leak was the most reported finding, included in 15/19 studies. The *International Consensus on Standardization of Data Collection for Complications Associated With Esophagectomy from the Esophagectomy Complications Consensus Group (ECCG)* was published in 2015 to better define and record perioperative complications associated with oesophagectomy and standardise international data [40]. These guidelines recommend that outcomes such as anastomotic leak rate, mortality, pulmonary complications and necrosis rates should be reported in any study reviewing outcomes in oesophagectomy. As many of the included studies were published prior to these guidelines, and this variation is to be expected.

In the results pertaining to the primary outcome, anastomotic leak is more varied. Although there was no statistically significant difference in *AL* rates between the methods, on analysis of raw data *JF* reconstruction showed the highest anastomotic leak rate at 18.1% (43/237), followed by *CI* at 12.4% (57/460) and *GP* at 8.9% (215/2403). League rank tables ranked *CI* worst in terms of *AL*. Existing literature on anastomotic leak with *JF* oesophageal reconstruction had reported rates ranging from 0 to 36% [13]. Traditional factors implicated in leakage rates include vascular supply and factors that affect the anastomosis, such as tension and pressure as well as systemic nutrition [41, 42]. A potential reason for higher rates of anastomotic leak rates in the *CI* group is because of a greater abundance of intestinal flora within the colon [27]. Doki et al.’s proposed theory is that intestinal flora may have a role in obstruction and prolonging healing which results in leakage [27]. Individual studies comparing *AL* with the reconstructive methods have also shown variable results. Luan et al. had a supercharged *JF AL* rate of 13.3% and *CI AL* rate of 75% [34]. Triboulet et al. have significantly higher *AL* rates in both *JF* and *CI* with rates of 32% and 40%, respectively [37]. This skewing of results is most likely due to the smaller number of patients within both the *JF* and *CI* groups, when compared to *GPU*. At 237 patients, the *JF* group was the smallest group within this analysis that looked et al. This suggests that at present this is the reconstructive method with the least experience in evolution. It is expected with increased use of these reconstructive techniques, more consistent results will be available. The location of anastomosis is a known risk factor for anastomotic leakage in the context of oesophagectomy with cervical anastomosis having a five times greater risk of leakage when compared to intrathoracic location [43]. A recent randomised control trial by van Workum et al. further supported this finding in the context of minimally invasive oesophagectomy [44]. Studies within the systematic review included both anastomotic techniques, with 8 studies using exclusively cervical anastomosis, 2 studies using intrathoracic, 5 using both and the rest unreported. The anastomotic location was most often decided by the location of the tumour and desired oncological resection, so therefore anastomosis location was not included in the analysis.

When comparing *JF* to *CI* as alternatives, there are reported advantages of both techniques. At present, both these techniques are usually reserved for recurrence or in salvage procedures when the stomach is not available. When compared to the colon, the jejunum is typically free of disease, has a luminal diameter similar in size to that of the oesophagus and has a decreased tendency to undergo senescent lengthening and therefore redundancy over time [13]. Furthermore, there are reports of superior peristalsis with *JF* [45]. Deficits in peristalsis can cause regurgitation or retention, which can in turn result in complications such as aspiration pneumonia or oesophagitis [34]. At present, *JF* is considered the more complex alternative when compared to *CI*, and it is often reserved as the ‘third line’. *JF* reconstruction traditionally has at least three bowel anastomosis sites and two microvascular sites anastomose requiring specialist microsurgeons. Previously, *JF* reconstruction was limited to short-segment reconstruction due to issues with its vasculature resulting in necrosis and higher mortality [34]. However with advances in microsurgical techniques and the use of supercharge techniques, *JF* has emerged as an appropriate option for total or thoracic oesophageal reconstruction [46].

The supercharge technique aims to augment the vascularity of the flap by using additional microvascular anastomosing of graft vessels to the recipient’s vessels. The technique was first described in 1946 by Longmire et al. [47]; however, the technique was initially not widely accepted due to its complex nature. Since the twenty-first century, it has regained popularity across microsurgical reconstructions. Supercharging is reported to improve the overall robustness of the grafts and reduce *AL* rates and further complications relating to graft ischaemia in oesophageal reconstruction [48]. Two of the nineteen studies included in our analysis reported the use of supercharged techniques. The majority of included studies did not report if the technique was used. The supercharge technique was used for *JF* in both studies. Doki et al. directly compare *SJF* and a supercharged *CI* conduit, while Luan et al. compare *SJF* to a standard *CI* [27, 34]. Both studies suggest that the *JF* performs better in terms on *AL*, stricture formations and length of stay [27, 34]. *GP* is not routinely performed with the supercharged procedure; however, this practice is currently changing. A prospective comparative study found that the supercharged cervical anastomosis for oesophagectomy (SAFE) procedure significantly reduced the postoperative complication rate and hospital stay [49].The recent SAFE (Supercharged Cervical Anastomosis for Esophagectomy) study examined the effects of supercharge techniques in the context of *GPU* reconstruction [49]. This prospective comparative study evaluated patients who underwent oesophagectomy with gastric reconstruction and cervical anastomosis for locally advanced oesophageal carcinoma. Patients were divided into two groups: (1) conventional cervical anastomosis was performed; (2) cervical anastomosis using the supercharged cervical anastomosis for oesophagectomy procedure was performed. Patients in group 2 experienced lower rates of *AL*, stricture rates and improved perfusion [49]. Venous congestion is most often the issue with these reconstructive methods, with a 26.5% improvement in perfusion observed in the SAFE study after venous super-drainage alone [49]. Multi-disciplinary planning is at the mainstay of ensuring success with these procedures. In the context of supercharging *GPU*, the short vessels or branches from the left gastroepiploic artery and vein from the greater curvature of the gastric tube closer to anastomosis are most often used and should be preserved. For revascularization at the neck, the cervical transverse artery, external jugular vein, anterior jugular vein, internal jugular vein and their branches should be preserved for anastomosis. When considering the future of complex oesophageal reconstructions and indeed cases of recurrence, the use of supercharging microvascular techniques is an obvious asset in all reconstructive methods.

There are several limitations to this study. Firstly, none of the studies included were randomised controlled trials. There are currently no randomised controlled trials comparing the described reconstructive methods. Furthermore, a number of studies included in this meta-analysis are over 20 years old and consequently, complication classification prior to the ECCG consensus will be haphazard. These older studies were performed before alternatives to the *GP* reconstructive method were more widely practised, and the use of more complex microsurgical techniques such as supercharging were used. The included studies were also diverse in reported patient and pathological characteristics. Four of the nineteen studies did not report on patient age, with a further eleven of the studies not commenting on the disease stage.

## Conclusion

In conclusion, the gastric conduit remains the cornerstone in reconstructive technique ab initio; however, when this conventional approach is not available, this study has demonstrated equipoise between *CI* and *JF*. With the introduction and refinement of superior microsurgical techniques, ongoing improvement in patient outcomes is expected in this field and facilitates the expansion of the surgeon’s armamentarium in the context of oesophagal reconstructions.
